# RAFT Polymerization of Styrene and Maleimide in the Presence of Fluoroalcohol: Hydrogen Bonding Effects with Classical Alternating Copolymerization as Reference

**DOI:** 10.3390/polym9030089

**Published:** 2017-03-03

**Authors:** Fangjun Yao, Qingqing Liu, Zhengbiao Zhang, Xiulin Zhu

**Affiliations:** Suzhou Key Laboratory of Macromolecular Design and Precision Synthesis, Jiangsu Key Laboratory of Advanced Functional Polymer Design and Application, State and Local Joint Engineering Laboratory for Novel Functional Polymeric Materials, College of Chemistry, Chemical Engineering and Materials Science, Soochow University, Suzhou 215123, China; 20144209220@stu.suda.edu.cn (F.Y.); lqq613677@163.com (Q.L.)

**Keywords:** alternating copolymerization, hydrogen bonding, *N*-phenylmaleimide, fluoroalcohol, reversible addition-fragmentation chain transfer (RAFT) copolymerization

## Abstract

The impacts of hydrogen bonding on polymerization behavior has been of interest for a long time; however, universality and in-depth understanding are still lacking. For the first time, the effect of hydrogen bonding on the classical alternating-type copolymerization of styrene and maleimide was explored. *N*-phenylmaleimide (N-PMI)/styrene was chosen as a model monomer pair in the presence of hydrogen bonding donor solvent 1,1,1,3,3,3-hexafluoro-2-propanol (HFIP), which interacted with N-PMI via hydrogen bonding. Reversible addition-fragmentation chain transfer polymerization (RAFT) technique was used to guarantee the “living” polymerization and thus the homogeneity of chain compositions. In comparison with the polymerization in non-hydrogen bonding donor solvent (toluene), the copolymerization in HFIP exhibited a high rate and a slight deviation from alternating copolymerization tendency. The reactivity ratios of N-PMI and St were revealed to be 0.078 and 0.068, respectively, while the reactivity ratios in toluene were 0.026 and 0.050. These interesting results were reasonably explained by using computer simulations, wherein the steric repulsion and electron induction by the hydrogen bonding between HFIP and N-PMI were revealed. This work first elucidated the hydrogen bonding interaction in the classical alternating-type copolymerization, which will enrich the research on hydrogen bonding-induced polymerizations.

## 1. Introduction

The highly polar solvent fluoroalcohol interacts with monomers via hydrogen bonding and has been frequently utilized in polymerization to facilitate polymerization and to regulate polymer microstructures [1,2,3,4,5]. The polar and bulky fluoroalcohol can interact with the polar substituents of monomer units such as methacrylates, vinyl esters, and acrylamides to induce syndiospecific radical polymerizations via steric repulsion [5]. Kakuchi and coworkers reported the synthesis of highly syndiotactic poly(methyl methacrylate) (PMMA) with very narrow polydispersity on the basis of the atom transfer radical polymerization (ATRP) with 1,1,1,3,3,3-hexafluoro-2-propanol (HFIP) as a highly polar solvent [2,4]. Takahara et al. cleverly used fluoroalcohol as a hydrogen bonding donor solvent to synthesize well-defined poly(sulfobetaine) brushes via surface-initiated atom transfer radical polymerization [6]. Hydrogen bonding created by this fluoroalcohol maintained affinity between polymers and sulfobetaine monomers; therefore, the homogeneous surface-initiated polymerization behavior could be significantly improved. Okamoto et al. speculated that the interactions between hydrogen bonding donor and hydrogen bonding receptor could form a monomer-activating effect due to the electron induction effects [7]. Our group reported that HFIP could be applied in controlled radical polymerization of 2-vinylpyridine (2VP) [8], 4-vinylpyridine (4VP) [9], and *N*-vinylpyrrolidone (NVP) [10] for better molecular weight, tacticity, and sequence control. Through elaborate NMR investigation, the monomer-activating effect was firstly proved by experimental evidence and computer simulation.

Poly(maleimide) and its derivatives are fascinating polymers because of their versatile applications in constructing surface functional polymers, polymer reagents, self-healing bio-based materials, high thermal resistance materials, and so on [11,12,13,14]. Meanwhile, due to the special structure of maleimide, the electron-deficiency vinyl double bonds usually give rise to unique polymerization behavior, such as a strong alternating cross propagation tendency with monomer-containing electron-rich vinyl double bonds. Therefore, maleimide was usually used as a special comonomer to create sequence-controlled polymers. Lutz et al. elegantly demonstrated the sequence-controlled macromolecules during ATRP by successive addition of a small amount of maleimide at a determined time [15]. By using this approach, tailored sequence-controlled polymers can be prepared by programmable incorporations of maleimide groups at desirable positions along the “living” chains. Moreover, the carbonyl groups neighboring the vinyl double bond can associate with a hydrogen bonding donor solvent, such as fluoroalcohol. Kamigaito et al. reported a pioneering work that fluorinated cumyl alcohol [PhC(CF_3_)_2_OH] could exert to construct a precise 1:2 sequence-regulated copolymer with limonene and *N*-phenylmaleimide (N-PMI) [5] by making use of the penultimate effect arising from hydrogen bonding.

Although the effects of hydrogen bonding on polymerization have been illustrated, the degree of the impact of the imposed hydrogen bonding—especially the monomer activity and copolymer composition—has rarely been clarified. To elucidate this, a polymerization system (comonomer pair) should be established as a comparison reference. In this work, the polymerization system was judiciously selected by using styrene (St)/maleimide as comonomer pair. It is well-known that maleimide has a high cross-propagation tendency with St to form a typical alternating copolymer, which can serve as the reference standard for investigating the effects of polymerization environments on polymerization behavior and the resultant polymers. Herein, to investigate how hydrogen bonding affects the classical alternating copolymerization and to what degree the hydrogen bonding affects the monomer activity and copolymer composition (i.e., the degree of deviation from the classical 1/1 composition), the copolymerization of maleimide and St in the presence of fluoroalcohol was explored. Reversible addition-fragmentation chain transfer polymerization (RAFT) technique was used for homogeneous chain compositions due to its “living” nature. This study will offer helpful results for understanding the effects of hydrogen bonding on polymerization, and might eventually direct the synthesis of novel biocompatible polymeric materials. 

## 2. Experiment

### 2.1. Materials

*N*-phenylmaleimide (N-PMI, 98%, Energy Chemical Co., Semnan, Iran) was recrystallized in toluene and methanol two times. Styrene (St, >99%, Chinasun Specialty Products Co. Ltd., Changshu, China) was deinhibited by percolating over a basic alumina column, and stored at −18 °C. 2-Cyanoprop-2-yl dithionaphthalenoate (CPDN) was synthesized by the method from Reference [16]. Azodiisobutyronitrile (AIBN, 99%, Energy Chemical Co.) was recrystallized in hot ethanol three times and concentrated by vacuum filtration. 1,1,1,3,3,3-Hexafluoro-2-propanol (HFIP, 99.5%, Jinan Weidu Chemical Co., Jinan, China), 1H,1H,5H-octafluoropentan-1-ol (99%, Alfa Aesar (China) Chemical Co. Ltd., Beijing, China), 2,2,3,3-tetrafluoro-1-propanol (99%, Energy Chemical Co.), and 2,2,2-trifluoroethanol (TFE, Aladdin Chemistry Co. Ltd., Shanghai, China) were used after eliminating a small quantity of impurity by distilling at reduced pressure. Toluene (99%, Shanghai Chemical Reagents Co., Shanghai, China), anisole (99%, Shanghai Chemical Reagents Co.), and isopropanol (>99%, Chinasun Specialty Products Co., Ltd.) were used as received.

### 2.2. Typical Procedures for RAFT Copolymerization of St and N-PMI in Various Solvent Systems

As for the molar ratio with [N-PMI]_0_/[St]_0_/[CPDN]_0_/[AIBN]_0_ = 250/250/2/1, N-PMI (0.747 g, 4.315 mmol), St (0.449 g, 4.315 mmol), RAFT agent (CPDN, 9.3 mg, 0.0345 mmol), initiator (AIBN, 2.8 mg, 0.0173 mmol), solvent (HFIP, 0.462 mL), and toluene (0.459 mL) were added into a dry and clean ampoule. Then, the mixture was deoxygenated through freeze–pump–thaw for three times. The ampoule was sealed by flame and bathed in a water pot under magnetic stirring at 40 °C. After a predesigned reaction time, the polymerization was stopped by putting the ampoule in iced water, and then the ampoule was opened at room temperature. The polymer was dissolved in tetrahydrofuran (THF) and was precipitated in cool methanol with stirring. After being filtered and dried in vacuum at 30 °C, the polymer was recovered and collected. The total conversion of copolymer was calculated with *m*[poly(N-PMI-*co*-St)]/[*m*(N-PMI)_0_ + *m*(St)_0_] × 100%, the *Đ* = *M*_w_/*M*_n_ values was calculated from the data from exclusion chromatography (SEC) (THF) with PS standards.

### 2.3. Characterization

The number-average molar mass (*M*_n_) and distributions of molecular weight (*Đ* = *M*_w_/*M*_n_) were determined by a size exclusion chromatography (SEC) TOSOH HLC-8320 instrument equipped with a refractive-index detector, with two columns of TSKgel Super Mutipore HZ-N (4.6 mm × 150 mm, 3 μm beads size) in series arrangement with molecular weight separations from 0.5 to 1.9 × 10^2^ kDa. THF was used as the eluent at a flow rate of 0.35 mL/min at 40 °C. Data obtained was performed by EcoSEC software, and PS standards were used to calculate the molecular weights. The ^1^H NMR spectrum of the precipitated copolymer was recorded on a Bruker nuclear magnetic resonance instrument (300 MHz) at room temperature, with tetramethylsilane (TMS) as the internal standard. CDCl_3_ (10–20 mg/mL) was used as solvent, and the ^1^H NMR spectra were referenced to δ 7.26 ppm. Elemental analysis (C, H, and N) were recorded with an EA1110 CHNO–S instrument. Differential scanning calorimetry (DSC) was executed with a TA Instruments DSC2010 with a heating/cooling rate of 20 °C·min^−1^ from 30 to 300 °C under a continuous nitrogen flow. It was calibrated by pure indium for enthalpy and temperature changes. Samples were held in standard aluminum pans. 

### 2.4. Computational Details

To determine the chemical relationship of hydrogen bonding between N-PMI and HFIP, the total theoretical calculations were executed at three parameter hybrid B3LYP density functional method with the extended basis set 6-311++G(d,p) implemented in the GAUSSIAN 09 package [17]. The molecular electrostatic potential (MEP) surface was gained from optimized geometry over Mulliken atomic charge of N-PMI and HFIP using the same software package. The shape of HOMO and LUMO orbitals were calculated by GaussView 5.0.9, including the visualization of optimized molecular structures [18]. The computer simulation on the microstructure of the copolymer was based on the semi-empirical quantum chemistry method with pm6 implemented in the GAUSSIAN 09 package.

## 3. Results and Discussion

**Figure 1:**
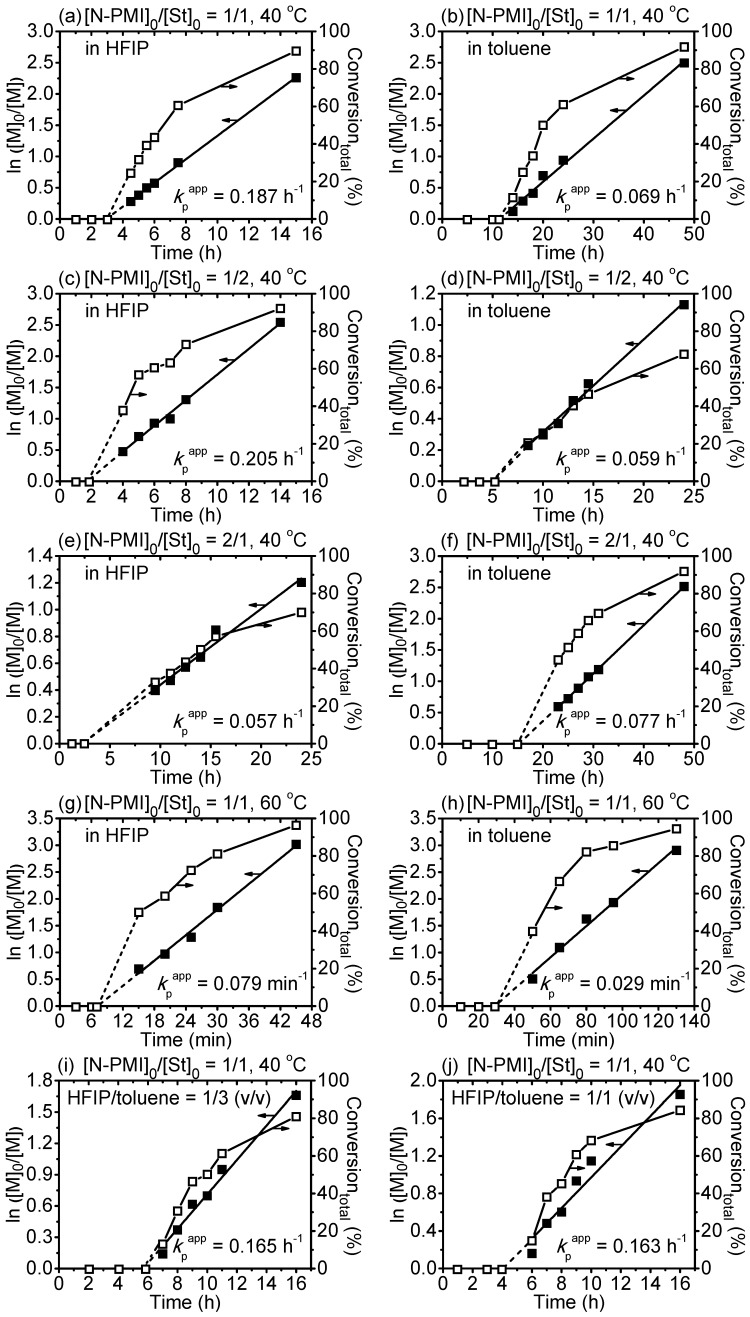

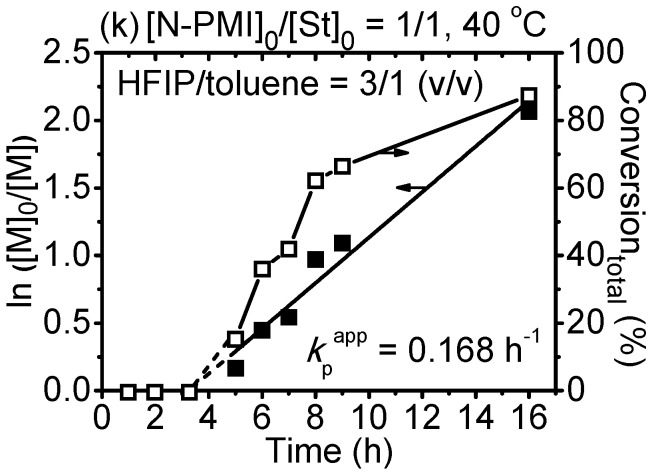
Polymerization kinetics of styrene (St) and *N*-phenylmaleimide (N-PMI) via reversible addition-fragmentation chain transfer (RAFT) copolymerization with different molar ratios, temperatures, and solvents. (**a**) in 1,1,1,3,3,3-hexafluoro-2-propanol (HFIP) and (**b**) in toluene: [N-PMI]_0_/[St]_0_/[CPDN]_0_/[AIBN]_0_ = 250/250/2/1, 40 °C; (**c**) in HFIP and (**d**) in toluene: [N-PMI]_0_/[St]_0_/[CPDN]_0_/[AIBN]_0_ = 167/333/2/1, 40°C; (**e**) in HFIP and (**f**) in toluene: [N-PMI]_0_/[St]_0_/[CPDN]_0_/[AIBN]_0_ = 333/167/2/1, 40 °C; (**g**) in HFIP and (**h**) in toluene: [N-PMI]_0_/[St]_0_/[CPDN]_0_/[AIBN]_0_ = 250/250/2/1, 60 °C; (**i**) in HFIP/toluene (HFIP/toluene = 1/3, *v*/*v*); (**j**) in HFIP/toluene (HFIP/toluene = 1/1, *v*/*v*); (**k**) in HFIP/toluene (HFIP/toluene = 3/1, *v*/*v*); (**i**–**k**): [N-PMI]_0_/[St]_0_/[CPDN]_0_/[AIBN]_0_ = 250/250/2/1, 40 °C. [N-PMI]_0_/[HFIP or toluene or HFIP/toluene]_0_ = 1/1. [M]_0_ and [M] were represented to the initial and current concentration of total monomer. AIBN: azodiisobutyronitrile; CPDN: 2-cyanoprop-2-yl dithionaphthalenoate.

To elucidate the hydrogen bonding effects on the polymerization, RAFT polymerizations under different molar ratios of N-PMI to St were implemented in HFIP and toluene, respectively. Moreover, because the temperature is usually a key factor in the intensity of hydrogen bonding, the polymerizations were also conducted under 40 and 60 °C for a more complete understanding. All the results are summarized and presented in Figure 1.

Figure 1 exhibits all the kinetic data of RAFT copolymerizations with different molar ratios in HFIP and toluene. All the polymerization kinetics conveyed linear plots, indicating the “living” nature of the polymerization. The “living” nature of the polymerization in both HFIP and toluene was also manifested by chain end analysis (Figure S1, Supplementary Materials), the increased molecular weights with conversion, as well as relatively narrow molecular weight distributions as shown in SEC traces in Figure S2 (Supplementary Materials). The “living” features of the polymerization ensured the homogenous chain compositions. It was noted that the copolymerization in HFIP showed shorter induction periods and higher polymerization rates than those in toluene. For example, for the [N-PMI]_0_/[St]_0_ = 1/1 at 40 °C, the induction period was about 3 h in HFIP with *k*_p_^app^ of 0.187 h^−1^ (Figure 1a), while it was about 10 h in toluene with 0.069 h^−1^ (Figure 1b). Furthermore, the polymerization in a mixture of HFIP and toluene showed similar phenomena. With increasing HFIP/toluene (*v*/*v*) (from 1/3 through 1/1 to 3/1), the induction periods were significantly reduced (Figure 1i–k). These results implied that the HFIP was probably associated with N-PMI via hydrogen bonding, which might alter the reactivity of N-PMI [8,9,10]. The hydrogen bonding interaction between HFIP and N-PMI units was explored by adding HFIP portion-wise into the copolymer in toluene. The changes of the signals from the protons of hydroxyl groups of HFIP can be easily found upon HFIP titrations (Figure S1), providing solid evidence for the hydrogen bonding interaction between maleimide and HFIP. This accelerating polymerization rate could also be observed in other hydrogen bonding donor solvents (as summarized in Table 1), such as 1H,1H,5H-octafluoro-1-pentanol, 2,2,2-trifluoroethanol, 2,2,3,3-tetrafluoro-1-propanol, etc. The acceleration of polymerization rate was because the introduction of fluoroalcohols may cause alterations of the electron cloud distribution of N-PMI through the formation of hydrogen bonding, which might cause a change in the reactivity of N-PMI. The RAFT polymerization of N-PMI and St at 60 °C also conveyed comparable “living” manners with respect to those at 40 °C. Meanwhile, the RAFT polymerization at 20 °C by using blue LED light as initiation source was implemented to investigate the influence of low temperature on the hydrogen bonding effects. The results summarized in Table 1 indicate that at 20 °C, the polymerization rate in HFIP was higher than those in toluene. Due to the different initiation types, the gap of the polymerization rate between HFIP and toluene in light-induced polymerizations was less significant as those AIBN-imitated polymerizations. However, at 20 °C, the Incorp_N-PMI_/Incorp_St_ in the HFIP system was much higher than that in toluene system, especially at high conversion. The reason can be ascribed to the stronger hydrogen bonding interaction between fluorinated alcohol with the carbonyl groups in the maleimide at lower temperature, which induced the greater incorporation of N-PMI via the penultimate model [5]. 

**Figure 2:**
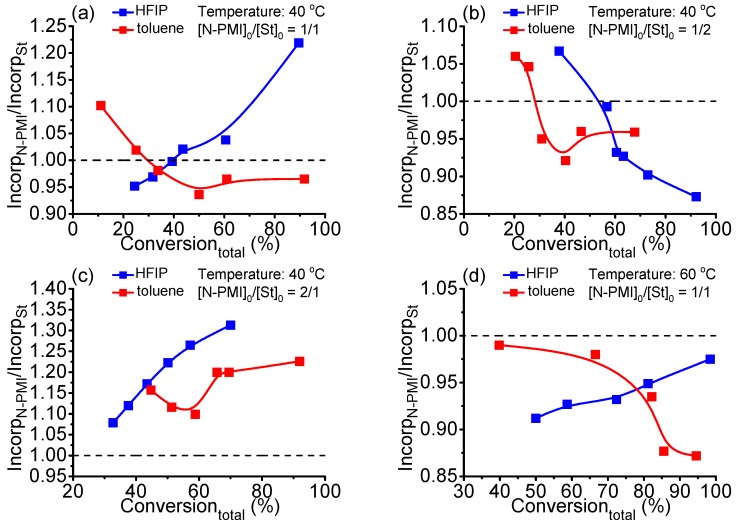
Incorp_N-PMI_/Incorp_St_ of resultant copolymer in HFIP and toluene. (**a**) [N-PMI]_0_/[St]_0_/[CPDN]_0_/[AIBN]_0_ = 250/250/2/1, 40 °C; (**b**) [N-PMI]_0_/[St]_0_/[CPDN]_0_/[AIBN]_0_ = 167/333/2/1, 40 °C; (**c**) [N-PMI]_0_/[St]_0_/[CPDN]_0_/[AIBN]_0_ = 333/167/2/1, 40 °C; (**d**) [N-PMI]_0_/[St]_0_/[CPDN]_0_/[AIBN]_0_ = 250/250/2/1, 60 °C; Incorp_N-PMI_/Incorp_St_ = (*c*_N_/*C*_N_)/*M*_N-PMI_/[1 − (cN/CN)] × *M*_st_, *c*_N_ is nitrogen content of copolymer, *C*_N_ is nitrogen content of N-PMI that refers to available data. The nitrogen content data from elemental analysis were characterized each for at least three times and averaged.

Figure 2 exhibits the dependence of incorporation ratio of N-PMI and St units (incorp_N-PMI_/incorp_St_) on total conversion of the above RAFT polymerizations. It was revealed from Figure 2 that with [N-PMI]_0_/[St]_0_ = 1/1 at 40 °C, the incorporation of N-PMI units in copolymers obtained with HFIP as solvent differed from those with toluene. In general, the polymerization in HFIP slightly deviated from the alternating polymerization tendency compared to those in toluene (Figure 2a). Changing the feed ratio of N-PMI and St—for example, [N-PMI]_0_/[St]_0_ = 1/2 (Figure 2b) and [N-PMI]_0_/[St]_0_ = 2/1 (Figure 2c)—the RAFT polymerization in toluene conveyed the alternating polymerization tendency. The polymerization in HFIP also moderately deviated more or less from the alternating polymerization tendency. As for the polymerization at 60 °C with [N-PMI]_0_/[St]_0_ = 1/1, the polymerization in toluene slightly deviated from the alternating copolymerization with conversion evolution. However, when using HFIP as solvent, the polymerization presented an alternating copolymerization tendency with the increase of conversion. These above results demonstrated that HFIP produced different polymerization behavior with respect to the toluene, indicating that HFIP might associate with N-PMI monomer and then alter the polymerization preference of N-PMI.

**Figure 3:**
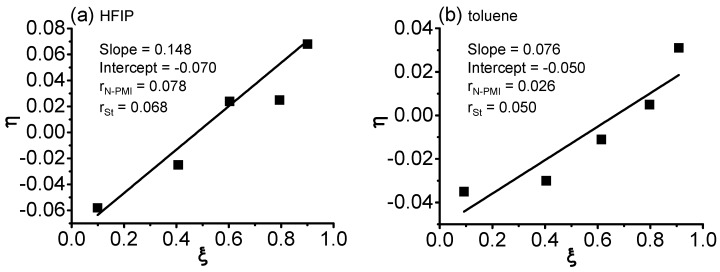
Reactivity ratios of N-PMI and St via Kelen–Tüdõs method for the RAFT polymerization at 40 °C. (**a**) HFIP as solvent (**b**) toluene as solvent.

The reactivity ratios of N-PMI and St were calculated by Kelen–Tüdõs method [19,20] in the polymerization at 40 °C with HFIP and toluene as solvent, respectively. The data are listed in Tables S1 and S2 (Supporting Information). As shown in Figure 3, the estimated reactivity ratios of N-PMI and St were 0.078 and 0.068 in fluoroalcohol, 0.026 and 0.050 in toluene, respectively. These results show an obvious an alternating copolymerization tendency (*r*_N-PMI_ < 1, *r*_St_ < 1, *r*_N-PMI_ × *r*_St_ ≈ 0). It was also noted that in HFIP, both *r*_N-PMI_ and *r*_St_ were slightly higher than those in toluene, denoting slight deviations from alternating copolymerization tendency. These results complied with those in Figure 2 that show that polymerizations in HFIP were slightly different than those in toluene. 

**Figure 4:**
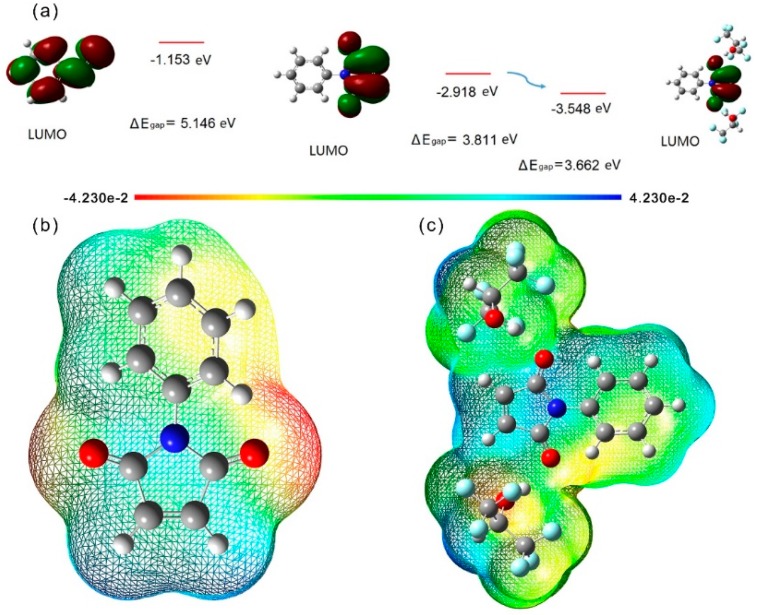
(**a**) Plot of frontier molecular orbitals of N-PMI and the N-PMI associated with HFIP; (**b**) Molecular electrostatic potential surface of N-PMI; (**c**) Molecular electrostatic potential surface of N-PMI and HFIP which connect with two hydrogen bonding in one representative position.

The above results indicated that the copolymerization of N-PMI and St in HFIP manifested a slightly different polymerization manner compared with those in toluene. The hydrogen bonding interaction between HFIP and N-PMI was possibly responsible for the different polymerization profile. To develop a deeper understanding of the hydrogen bonding effects on the N-PMI and the subsequent copolymerization, computer simulation was used [21] based on the three parameter hybrid B3LYP density functional method with the extended basis set 6-311++G(d,p) implemented in the GAUSSIAN 09 package. According to the simulation structure in Figure S3, the Mulliken charge of pivotal atoms in N-PMI all revealed meaningful changes when N-PMI interacted with HFIP. These results implied that the electron cloud distribution was altered by the introduction of hydrogen bonding [8,10]. To clearly elucidate the role of hydrogen bonding in the polymerization, molecular orbital based on computer simulation was used [22], simulating the lowest unoccupied molecular orbital (LUMO) and highest occupied molecular orbital (HOMO). The larger frontier orbital gap implies the lower chemical reactivity of the molecular [23,24]. According to Figure 4a, Δ*E*_gap_ shows a decrement when HFIP associated with N-PMI via hydrogen bonding. Therefore, this association of N-PMI with HFIP could be considered as a kind of activation process, making monomers more easily to accept electrons (i.e., being more easily attacked by initiating radicals or propagating radicals). This effect agreed with the results of Figure 1, wherein the induction period was shortened and the polymerization rate was elevated by using HFIP as solvent instead of toluene. Furthermore, electrostatic potential could be exhibited clearly by molecular electrostatic potential surface as illustrated in Figure 4b,c. Different colors represent different values for electrostatic potential; the potential decreased in the order blue > green > yellow > orange > red. Blue implies that the regions of electrostatic potential were positive, green represents less positive, and red means negative regions. It can be found that the molecular electrostatic potential surface of N-PMI in the absence of hydrogen bonding interaction showed a difference with the presence of hydrogen bonding interaction. The obvious changes of electrostatics potential of N-PMI help in understanding how hydrogen bonding affects the monomer reactivity.

**Figure 5:**
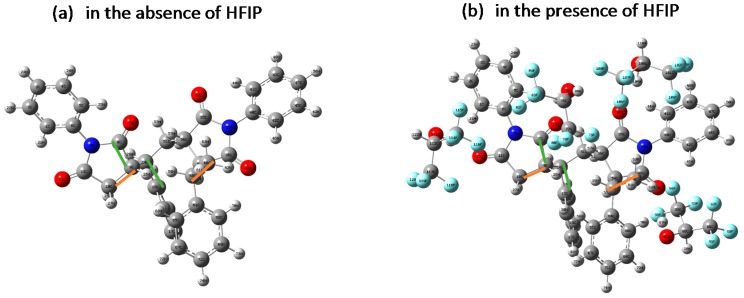
Computer simulation of tetrad alternating chain (N-PMI-St-N-PMI-St) conformation by using semi-empirical quantum chemistry method with pm6 implemented in the GAUSSIAN 09 package. (**a**) In the presence of HFIP; (**b**) In the absence of HFIP.

The copolymers from RAFT polymerizations with HFIP or toluene as solvent were measured by DSC in Table 2 and Figure S4; the results indicated that with almost identical molecular weights, the copolymers obtained from the HFIP system displayed slightly lower glass transition temperatures (*T*_g_) compared to those with toluene or anisole as solvent. The difference of *T*_g_ was possibly owing to the different microstructures of polymers obtained in HFIP and toluene. It has been well illustrated that the hydrogen bonding interaction between the bulky fluoroalcohol and monomer would create stereo-regulation of the resultant polymers [25]. In this work, the N-PMI monomer was associated with bulky HFIP, which would finally affect the stereo-regulation of N-PMI units; i.e., the N-PMI unit associated with HFIP would twist or bend from the neighboring N-PMI to lower the potential energy of the polymer chain. These assumptions were validated by using computer simulation, as shown in Figure 5. The results indicated that in the presence of HFIP, the dihedral angle between the plane of 10C–9C and 58C–60C (orange bar) changed from 117.62° (Figure 5a) to 132.38° (Figure 5b), implying some degree of distortion of the chain conformation. On the other hand, the dihedral angle between the plane of 8C–9C and 14C–15C (green bar) changed from 174.28° (Figure 5a) to 179.79° (Figure 5b), implying some degree of bend of the chain conformation. These results proved that the hydrogen bonding could change the resultant chain conformation to some degree, which might cause a change in chain entanglement and packing, and finally result in the different *T*_g_. In general, the polymer chain under hydrogen bonding conveyed more twisted and bended conformation, causing difficulty in chain packing and thus lower *T*_g_. Moreover, as demonstrated by Kamigaito et al. [5], one HFIP molecule can associate with two N-PMI monomers via hydrogen bonding interaction; as a consequence, the reactivity ratio of maleimide monomer and its comonomer was significantly changed and the unit sequence thus had more two consecutive N-PMI units (i.e., –(N-PMI)–(N-PMI)–). In the current work, since the St/N-PMI comonomer pair had a strong alternating copolymer tendency, the hydrogen bonding could not significantly alter the alternating properties. However, some microstructures of the unit sequence probably changed in a moderate manner due to hydrogen bonding interaction; i.e., slightly more –(N-PMI)–(N-PMI)– microstructures might have resulted. These different microstructures—including unit sequence and stereoregularity—then contributed to the difference of the properties, including the *T*_g_. These results implied that the polymerization induced by hydrogen bonding produced polymers with slightly changed compositions and thus the moderately different properties. Meaningfully, the results also opened a new way to tune the polymer properties by using hydrogen bonding effects in the polymerization. 

## 4. Conclusions

RAFT copolymerization of N-PMI and St was implemented in the hydrogen bonding donor solvent HFIP. The polymerization rate of the RAFT copolymerization was significantly elevated in HFIP compared with those in toluene. The evolution of copolymer composition as well as the calculations of reactive ratios demonstrated that the copolymerization in HFIP slightly deviated from the alternating copolymerization tendency. The hydrogen bonding interactions between N-PMI and HFIP were confirmed by using computer simulations, which also afforded reasonable explanations of the polymerization results. DSC results indicated that the glass transition temperature of the copolymer obtained with HFIP as solvent was different from that with toluene as solvent. This work extends the research on hydrogen bonding-induced polymerizations and endows a new way to finely tune the polymer property by the introduction of non-covalent interaction such as hydrogen bonding. 

## Figures and Tables

**Table 1:** RAFT polymerization of N-PMI and St in different solvents ^a^.

Solvent	Time (h)	Conv_total_ (%)	*M*_n_ (kDa)	*Ð*	Incorp_N-PMI_/Incorp_St_
1H,1H,5H-Octafluoropentan-1-ol	4.0	34.8	16.7	1.17	0.931
1H,1H,5H-Octafluoropentan-1-ol	8.0	96.1	26.7	1.67	1.070
2,2,3,3-Tetrafluoro-1-propanol	7.0	32.7	12.1	1.62	0.923
2,2,3,3-Tetrafluoro-1-propanol	11.5	69.2	30.4	1.62	1.034
Anisole	11.5	30.9	11.8	1.67	1.087
Anisole	22.0	63.7	25.4	1.42	0.963
2,2,2-Trifluoroethanol	5.0	47.4	18.5	1.21	0.909
2,2,2-Trifluoroethanol	11.5	81.4	27.8	1.35	1.023
Isopropanol	8.0	16.9	5.4	1.42	0.894
Isopropanol	36.0	81.9	17.0	2.90	0.949
HFIP ^b^	1.5	65.2	25.7	1.25	0.848
HFIP ^b^	3.0	82.0	35.8	1.43	1.380
Toluene ^b^	3.0	59.3	23.3	1.30	0.771
Toluene ^b^	6.0	78.1	38.9	1.21	0.716

^a^ [N-PMI]_0_/[St]_0_/[CPDN]_0_/[AIBN]_0_ = 250/250/2/1, 40 °C. Data of *M*_n_ and *Ð* were obtained from size exclusion chromatography (SEC) (tetrahydrofuran, THF) with PS as standards. Incorp_N-PMI_/Incorp_St_ = (*c*_N_/*C*_N_)/*M*_N-PMI_/[1 − (cN/CN)] × *M*_st_, wherein the *c*_N_ is the nitrogen content of copolymer, and *C*_N_ is nitrogen content of N-PMI. The *c*_N_ of each sample by elemental analysis were characterized three times and averaged; ^b^ [N-PMI]_0_/[St]_0_/[CPDN]_0_ = 250/250/2, 20 °C, blue LED-induced RAFT polymerization.

**Table 2:** Glass transition temperature of N-PMI/St copolymers obtained with different solvents.

Solvent	Time (h)	Temp. (°C)	[N-PMI]_0_/[St]_0_	*M*_n_ (kDa)	*Ð*	Incorp_N-PMI_/Incorp_St_	*T*_g_ (°C)
HFIP	5.5	40	1:1	11.5	1.38	1.087:1	204.0
Anisole	11.5	40	1:1	11.8	1.67	0.998:1	211.3
HFIP	9.0	40	1:1	21.4	1.38	1.167:1	208.2
Toluene	24.0	40	1:1	21.3	1.48	0.965:1	219.8
HFIP	4.0	40	1:2	12.1	1.21	1.067:1	187.9
Toluene	10.0	40	1:2	12.1	1.38	1.046:1	202.5
HFIP	24.0	40	2:1	22.7	1.53	1.313:1	183.9
Toluene	29.0	40	2:1	22.6	1.39	1.200:1	213.7
HFIP	0.75	60	1:1	26.9	1.29	0.975:1	211.3
Toluene	1.33	60	1:1	27.4	1.27	0.935:1	212.6

## Supplementary Materials

The following are available online at www.mdpi.com/2073-4360/9/3/89/s1. Figure S1: (a) ^1^H NMR spectrum of the copolymer of *N*-phenylmaleimide (N-PMI) and styrene (St) (*M*_n_,_SEC_ = 18.5 kDa, *Ɖ* = 1.35). CDCl_3_ was applied as the solvent. Copolymerization conditions: [N-PMI]_0_/[St]_0_/[CPDN]_0_/[AIBN]_0_ = 250/250/2/1, [HFIP]_0_/[N-PMI]_0_ = 1.0/1.0, St = 0.494 mL, time = 7.5 h, conversion_total_ = 60.6%; temperature = 40 °C. (b) ^1^H NMR spectrum of the titration adding 1,1,1,3,3,3-hexafluoro-2-propanol (HFIP) portion-wise into the toluene which had dissolved the N-PMI and St copolymers over certain time. Twenty milligrams of N-PMI-*co*-St (the same sample set above) was dissolved in 30 µL toluene, and titrated 15 µL HFIP for each time. Fifteen microliters of HFIP (HFIP/toluene = 1/2, *v/v*), 3.63 ppm; 30 µL of HFIP (HFIP/toluene = 1/1, *v/v*), 3.72 ppm; 45 µL of HFIP (HFIP/toluene = 3/2, *v/v*), 3.81 ppm; 60 µL of HFIP (HFIP/toluene = 2/1, *v/v*), 3.84 ppm. AIBN: azodiisobutyronitrile; CPDN: 2-cyanoprop-2-yl dithionaphthalenoate. Figure S2: Size exclusion chromatography (SEC) traces of reversible addition-fragmentation chain transfer (RAFT) copolymerization of N-PMI and St. (a) in HFIP and (b) in toluene: [N-PMI]_0_/[St]_0_/[CPDN]_0_/[AIBN]_0_ = 250/250/2/1, 40 °C; (c) in HFIP and (d) in toluene: [N-PMI]_0_/[St]_0_/[CPDN]_0_/[AIBN]_0_ = 167/333/2/1, 40 °C; (e) in HFIP and (f) in toluene: [N-PMI]_0_/[St]_0_/[CPDN]_0_/[AIBN]_0_ = 333/167/2/1, 40 °C; (g) in HFIP and (h) in toluene: [N-PMI]_0_/[St]_0_/[CPDN]_0_/[AIBN]_0_ = 250/250/2/1, 60 °C; (i) in HFIP/toluene (HFIP/toluene = 1/3, *v/v*); (j) in HFIP/toluene (HFIP/toluene = 1/1, *v/v*); (k) in HFIP/toluene (HFIP/toluene = 3/1, *v/v*). (i–k): [N-PMI]_0_/[St]_0_/[CPDN]_0_/[AIBN]_0_ = 250/250/2/1, 40 °C. [N-PMI]_0_/[HFIP or toluene or HFIP/toluene]_0_ = 1/1. Figure S3: (a) Molecular structure of N-PMI. (b) Molecular structure of N-PMI and HFIP which connect with two hydrogen bonding in one representative position. Figure S4: Differential scanning calorimetry (DSC) thermograms of N-PMI and St copolymers obtained in HFIP and toluene, respectively. DSC runs with a heating/cooling rate of 20 °C min^-1^ from 30 to 300 °C under a continuous nitrogen flow. Table S1: Data for calculating reactivity ratios of N-PMI and St with HFIP as solvent. Table S2: Data for calculating reactivity ratios of N-PMI and St with toluene as solvent.
