# Supplementary material for: RAFT Polymerization of Styrene and Maleimide in the Presence of Fluoroalcohol: Hydrogen Bonding Effects with Classical Alternating Copolymerization as Reference
Source: Polymers (Basel). 2017 Mar 3;9(3):89. doi: 10.3390/polym9030089 (PMC6432049; doi:10.3390/polym9030089)
Supplement: Supplementary file 1 [file polymers-09-00089-s001.pdf]

Supporting Information for

RAFT Polymerization of Styrene and Maleimide in the Presence of Fluoroalcohol: Hydrogen Bonding Effects with Classical Alternating Copolymerization as Reference

Fangjun Yao, Qingqing Liu, Zhengbiao Zhang*, and Xiulin Zhu*

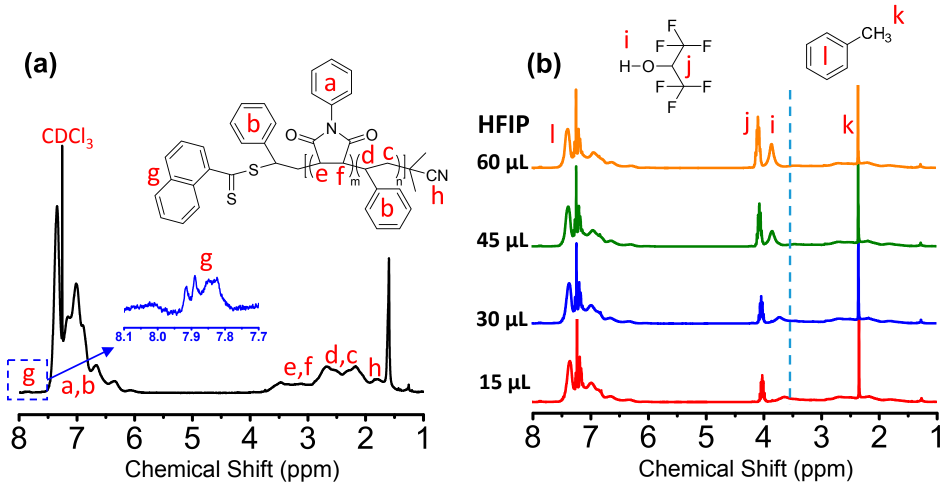

Figure S1. (**a**) ^{1}H NMR spectrum of the copolymer of *N*-phenylmaleimide (N-PMI) and styrene (St) ($M_{n,SEC}$ = 18.5 kDa, $Đ$ = 1.35). $CDCl_3$ was applied as the solvent. Copolymerization conditions: $[N\text{-}PMI]_0/[St]_0/[CPDN]_0/[AIBN]_0$ = 250/250/2/1, $[HFIP]_0/[N\text{-}PMI]_0$ = 1.0/1.0, St = 0.494 mL, time = 7.5 h, $conversion_{total}$ = 60.6%; temperature = 40 °C. (**b**) ^{1}H NMR spectrum of the titration adding 1,1,1,3,3,3-hexafluoro-2-propanol (HFIP) portion-wise into the toluene which had dissolved the N-PMI and St copolymers over certain time. Twenty milligrams of N-PMI-*co*-St (the same sample set above) was dissolved in 30 μL toluene, and titrated 15 μL HFIP for each time. Fifteen microliters of HFIP (HFIP/toluene = 1/2, *v*/*v*), 3.63 ppm; 30 μL of HFIP (HFIP/toluene = 1/1, *v*/*v*), 3.72 ppm; 45 μL of HFIP (HFIP/toluene = 3/2, *v*/*v*), 3.81 ppm; 60 μL of HFIP (HFIP/toluene = 2/1, *v*/*v*), 3.84 ppm. AIBN: azodiisobutyronitrile; CPDN: 2-cyanoprop-2-yl dithionaphthalenoate.

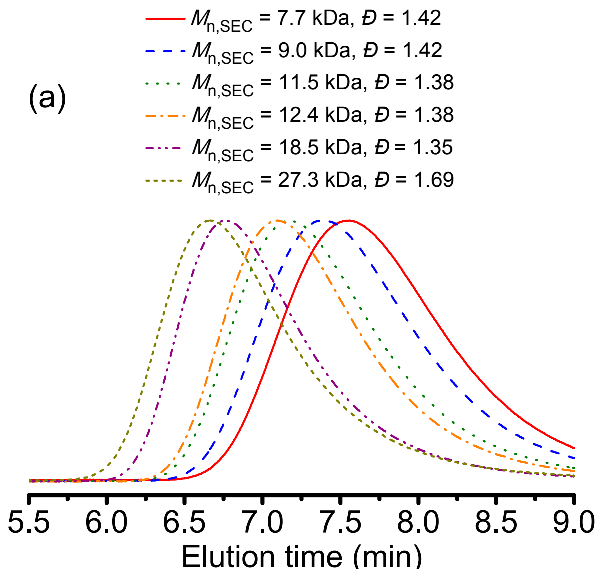

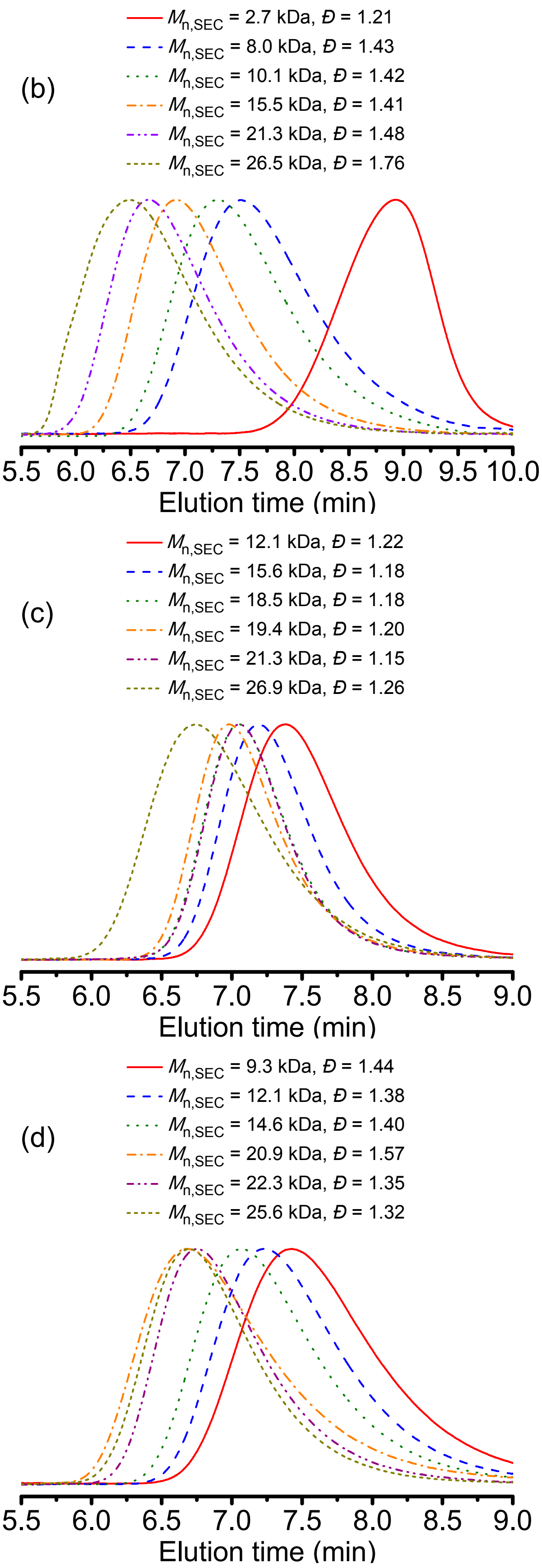
(b)
$M_{n,SEC}$ = 2.7 kDa, Đ = 1.21
$M_{n,SEC}$ = 8.0 kDa, Đ = 1.43
$M_{n,SEC}$ = 10.1 kDa, Đ = 1.42
$M_{n,SEC}$ = 15.5 kDa, Đ = 1.41
$M_{n,SEC}$ = 21.3 kDa, Đ = 1.48
$M_{n,SEC}$ = 26.5 kDa, Đ = 1.76
5.5 6.0 6.5 7.0 7.5 8.0 8.5 9.0 9.5 10.0
Elution time (min)
(c)
$M_{n,SEC}$ = 12.1 kDa, Đ = 1.22
$M_{n,SEC}$ = 15.6 kDa, Đ = 1.18
$M_{n,SEC}$ = 18.5 kDa, Đ = 1.18
$M_{n,SEC}$ = 19.4 kDa, Đ = 1.20
$M_{n,SEC}$ = 21.3 kDa, Đ = 1.15
$M_{n,SEC}$ = 26.9 kDa, Đ = 1.26
5.5 6.0 6.5 7.0 7.5 8.0 8.5 9.0
Elution time (min)
(d)
$M_{n,SEC}$ = 9.3 kDa, Đ = 1.44
$M_{n,SEC}$ = 12.1 kDa, Đ = 1.38
$M_{n,SEC}$ = 14.6 kDa, Đ = 1.40
$M_{n,SEC}$ = 20.9 kDa, Đ = 1.57
$M_{n,SEC}$ = 22.3 kDa, Đ = 1.35
$M_{n,SEC}$ = 25.6 kDa, Đ = 1.32
5.5 6.0 6.5 7.0 7.5 8.0 8.5 9.0
Elution time (min)

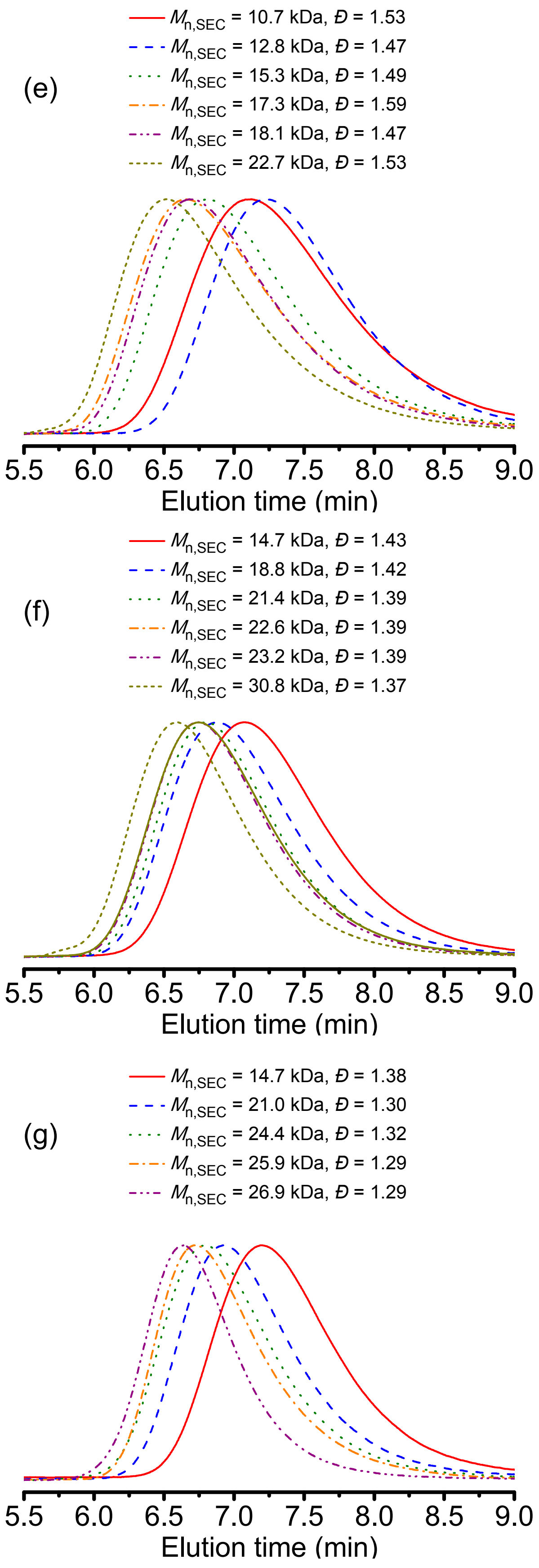

(e)
$M_{n,SEC}$ = 10.7 kDa, Đ = 1.53
$M_{n,SEC}$ = 12.8 kDa, Đ = 1.47
$M_{n,SEC}$ = 15.3 kDa, Đ = 1.49
$M_{n,SEC}$ = 17.3 kDa, Đ = 1.59
$M_{n,SEC}$ = 18.1 kDa, Đ = 1.47
$M_{n,SEC}$ = 22.7 kDa, Đ = 1.53
5.5 6.0 6.5 7.0 7.5 8.0 8.5 9.0
Elution time (min)
(f)
$M_{n,SEC}$ = 14.7 kDa, Đ = 1.43
$M_{n,SEC}$ = 18.8 kDa, Đ = 1.42
$M_{n,SEC}$ = 21.4 kDa, Đ = 1.39
$M_{n,SEC}$ = 22.6 kDa, Đ = 1.39
$M_{n,SEC}$ = 23.2 kDa, Đ = 1.39
$M_{n,SEC}$ = 30.8 kDa, Đ = 1.37
5.5 6.0 6.5 7.0 7.5 8.0 8.5 9.0
Elution time (min)
(g)
$M_{n,SEC}$ = 14.7 kDa, Đ = 1.38
$M_{n,SEC}$ = 21.0 kDa, Đ = 1.30
$M_{n,SEC}$ = 24.4 kDa, Đ = 1.32
$M_{n,SEC}$ = 25.9 kDa, Đ = 1.29
$M_{n,SEC}$ = 26.9 kDa, Đ = 1.29
5.5 6.0 6.5 7.0 7.5 8.0 8.5 9.0
Elution time (min)

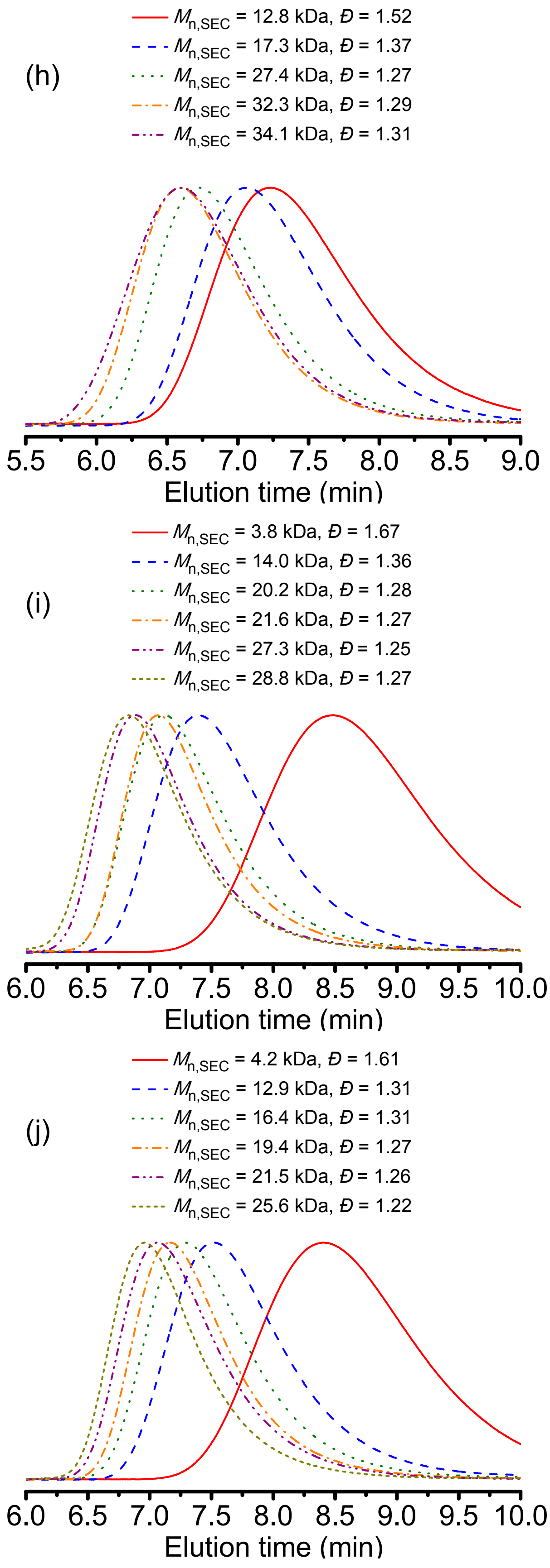
(h)
Mn,SEC = 12.8 kDa, Đ = 1.52
Mn,SEC = 17.3 kDa, Đ = 1.37
Mn,SEC = 27.4 kDa, Đ = 1.27
Mn,SEC = 32.3 kDa, Đ = 1.29
Mn,SEC = 34.1 kDa, Đ = 1.31
5.5 6.0 6.5 7.0 7.5 8.0 8.5 9.0
Elution time (min)
(i)
Mn,SEC = 3.8 kDa, Đ = 1.67
Mn,SEC = 14.0 kDa, Đ = 1.36
Mn,SEC = 20.2 kDa, Đ = 1.28
Mn,SEC = 21.6 kDa, Đ = 1.27
Mn,SEC = 27.3 kDa, Đ = 1.25
Mn,SEC = 28.8 kDa, Đ = 1.27
6.0 6.5 7.0 7.5 8.0 8.5 9.0 9.5 10.0
Elution time (min)
(j)
Mn,SEC = 4.2 kDa, Đ = 1.61
Mn,SEC = 12.9 kDa, Đ = 1.31
Mn,SEC = 16.4 kDa, Đ = 1.31
Mn,SEC = 19.4 kDa, Đ = 1.27
Mn,SEC = 21.5 kDa, Đ = 1.26
Mn,SEC = 25.6 kDa, Đ = 1.22
6.0 6.5 7.0 7.5 8.0 8.5 9.0 9.5 10.0
Elution time (min)

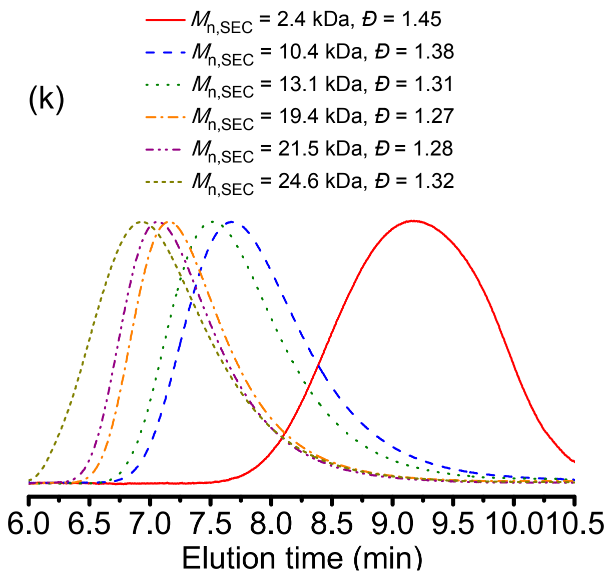

Figure. S2 Size exclusion chromatography (SEC) traces of reversible addition-fragmentation chain transfer (RAFT) copolymerization of N-PMI and St. (**a**) in HFIP and (**b**) in toluene: $[N\text{-}PMI]_0/[St]_0/[CPDN]_0/[AIBN]_0$ = 250/250/2/1, 40 °C; (**c**) in HFIP and (**d**) in toluene: $[N\text{-}PMI]_0/[St]_0/[CPDN]_0/[AIBN]_0$ = 167/333/2/1, 40 °C; (**e**) in HFIP and (**f**) in toluene: $[N\text{-}PMI]_0/[St]_0/[CPDN]_0/[AIBN]_0$ = 333/167/2/1, 40 °C; (**g**) in HFIP and (**h**) in toluene: $[N\text{-}PMI]_0/[St]_0/[CPDN]_0/[AIBN]_0$ = 250/250/2/1, 60 °C; (**i**) in HFIP/toluene (HFIP/toluene = 1/3, *v*/*v*); (**j**) in HFIP/toluene (HFIP/toluene = 1/1, *v*/*v*); (**k**) in HFIP/toluene (HFIP/toluene = 3/1, *v*/*v*). (i–k): $[N\text{-}PMI]_0/[St]_0/[CPDN]_0/[AIBN]_0$ = 250/250/2/1, 40 °C. $[N\text{-}PMI]_0$/[HFIP or toluene or HFIP/toluene]$_0$ = 1/1.

Table S1. Data for calculating reactivity ratios of N-PMI and St with HFIP as solvent [a].

$[N\text{-}PMI]_0/[St]_0$	$M_{N\text{-}PMI}$	M_{St}	$m_{N\text{-}PMI}$	m_{St}	x	y	η	ξ
30/100	0.231	0.769	0.581	0.419	0.300	0.834	-0.058	0.099
80/100	0.444	0.556	0.613	0.387	0.800	0.952	-0.025	0.407
100/80	0.556	0.444	0.636	0.364	1.250	1.051	0.024	0.603
100/50	0.667	0.333	0.638	0.362	2.000	1.060	0.025	0.794
100/30	0.769	0.231	0.675	0.325	3.333	1.250	0.068	0.900

Table S2. Data for calculating reactivity ratios of N-PMI and St with toluene as solvent [a].

$[N\text{-}PMI]_0/[St]_0$	$M_{N\text{-}PMI}$	M_{St}	$m_{N\text{-}PMI}$	m_{St}	x	Y	η	ξ
30/100	0.231	0.769	0.595	0.405	0.300	0.885	-0.035	0.092
80/100	0.444	0.556	0.610	0.390	0.800	0.940	-0.03	0.404
100/80	0.556	0.444	0.612	0.388	1.250	0.977	-0.011	0.614
100/50	0.667	0.333	0.627	0.377	2.000	1.013	0.005	0.797
100/30	0.769	0.231	0.650	0.350	3.333	1.116	0.031	0.908

[a] Copolymers were obtained from various monomer feed with $[N\text{-}PMI]_0/[St]_0$ = 30/100, 80/100, 100/80, 100/50, 100/30 in HFIP and toluene, respectively, 40 °C, and collected under the total conversion of 30%. $M_{N\text{-}PMI}$ and M_{St} refer to the feed compositions of the N-PMI and St, respectively. $m_{N\text{-}PMI}$ and m_{St} refer to the respective N-PMI and St unit compositions in the copolymer, calculated based on the nitrogen content from elemental analysis. $m_{N\text{-}PMI} = (c_N/C_N)/M_{N\text{-}PMI}$, $m_{St} = 1 - m_{N\text{-}PMI}$, c_N is nitrogen content of copolymer, C_N is nitrogen content of N-PMI. The nitrogen content data of each sample by elemental analysis were characterized three times and averaged. x, y, η, and ξ were calculated according to Kelen–Tüdõs method, and used for further calculations.

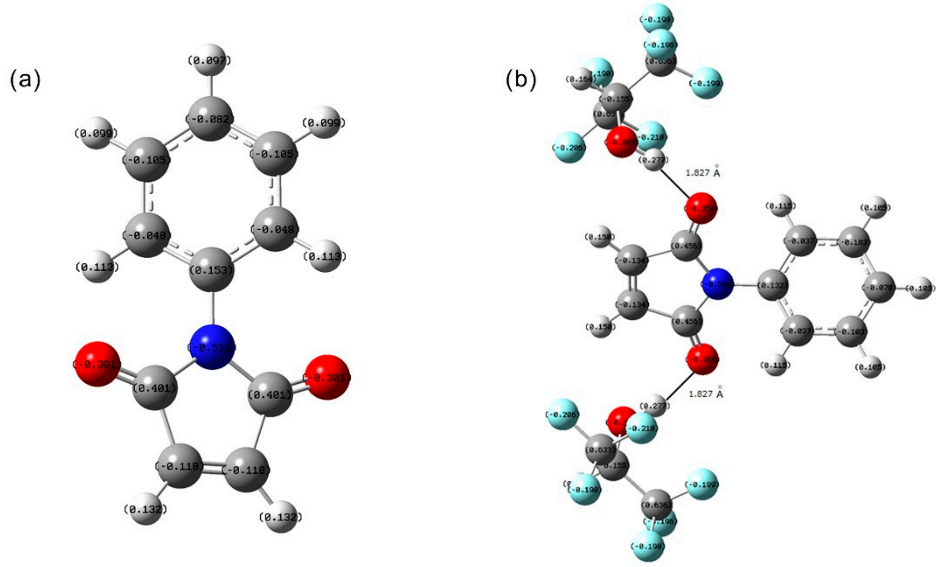

Figure S3. (**a**) Molecular structure of N-PMI. (**b**) Molecular structure of N-PMI and HFIP which connect with two hydrogen bonding in one representative position.

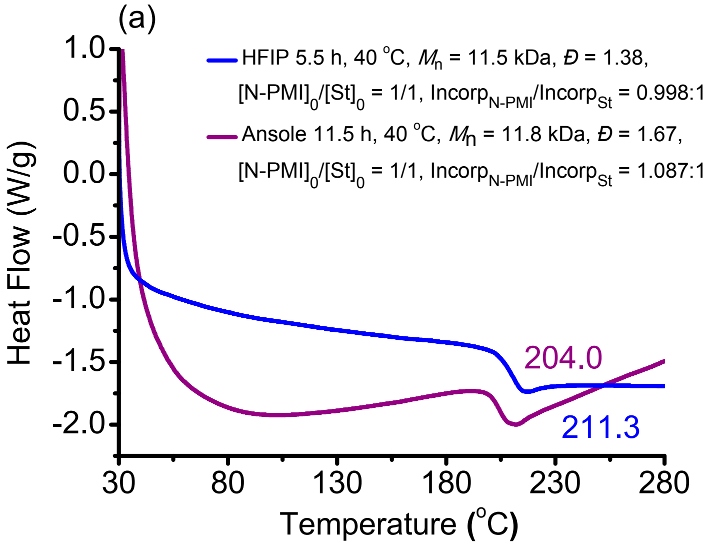

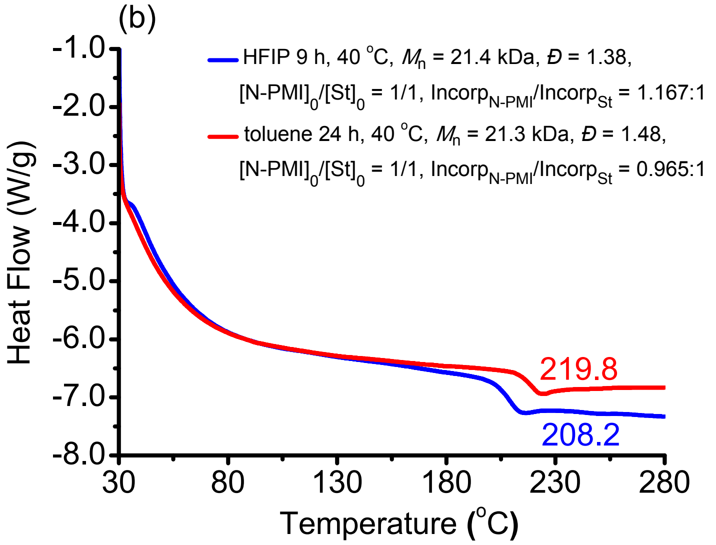

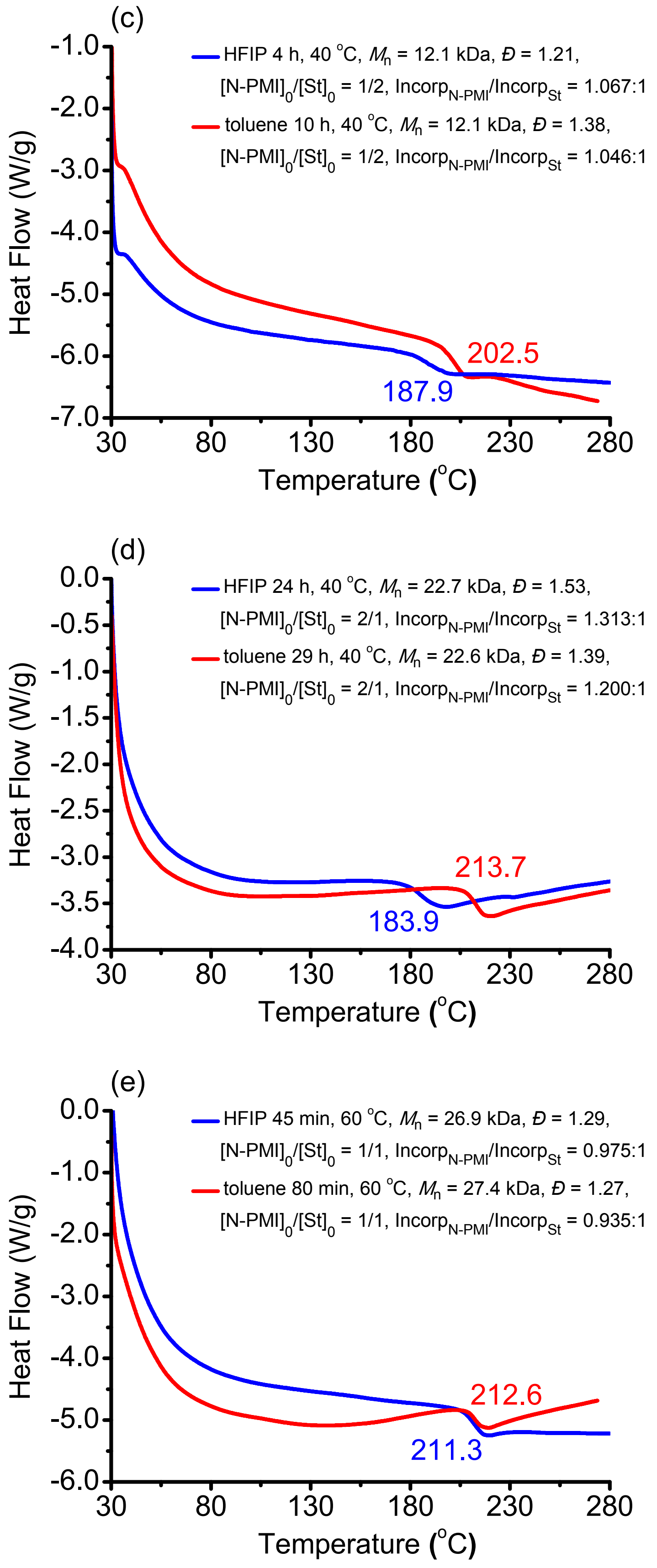

Figure. S4 Differential scanning calorimetry (DSC) thermograms of N-PMI and St copolymers obtained in HFIP and toluene, respectively. DSC runs with a heating/cooling rate of 20 °C min^{-1} from 30 to 300 °C under a continuous nitrogen flow.
